# Prodigiosin protects bone marrow-derived mesenchymal stem cells from hypoxia-induced apoptosis by modulating HIF-1α/YAP-associated signaling

**DOI:** 10.3389/fsysb.2026.1947639

**Published:** 2026-10-29

**Authors:** Yang Liu, Haixia Xu, ZhiHao Li, Lixin Zhu

**Affiliations:** 1 Department of Spine Orthopedics, Zhujiang Hospital, Southern Medical University, Guangzhou, China; 2 Department of Orthopedics, Jingzhou Hospital Affiliated to Yangtze University, Jingzhou, Hubei, China

**Keywords:** bone marrow-derived mesenchymal stem cells, HIF-1α, hypoxia-induced apoptosis, prodigiosin, YAP

## Abstract

**Introduction:**

Bone marrow-derived mesenchymal stem cells (BMSCs) are widely used in regenerative medicine, but their poor survival in hypoxic microenvironments limits their therapeutic efficacy. Here, we investigated whether Prodigiosin protects BMSCs against CoCl2-induced hypoxic injury and explored the involvement of HIF-1α/YAP-associated signaling.

**Methods:**

BMSCs were cultured and phenotyped by flow cytometry. Hypoxia was induced with CoCl (100 μM, 24 h). Cells were treated with Prodigiosin (20 nM) and/or Verteporfin (0.5 μM), or transfected with HIF-1α siRNA. Viability was measured by CCK-8; apoptosis by Annexin V/PI and DAPI; ROS by DHE; and protein expression by Western blot. Data were analyzed by ANOVA or unpaired t-test (P < 0.05).

**Results and Discussion:**

CoCl2 exposure reduced BMSCs viability, increased apoptosis, promoted reactive oxygen species (ROS) accumulation, and induced HIF-1α expression. These changes were accompanied by a decrease in the p-YAP/YAP ratio and increased expression of Bax and active caspase-3. Prodigiosin treatment alleviated CoCl?-induced cytotoxicity, suppressed ROS accumulation and apoptosis, and partially restored the p-YAP/YAP ratio. In addition, both the YAP inhibitor Verteporfin and HIF-1α knockdown reduced hypoxia-induced apoptosis, supporting the involvement of HIF-1α/YAP-associated signaling in BMSC injury under hypoxic stress. Together, these results suggest that Prodigiosin protects BMSCs from hypoxia-induced apoptosis, at least in part by modulating HIF-1α/YAP-related signaling, and may help improve stem cell survival in hypoxic regenerative environments.

## Introduction

Bone marrow-derived mesenchymal stem cells (BMSCs) have emerged as promising candidates for regenerative medicine owing to their self-renewal capacity, multilineage differentiation potential, and immunomodulatory properties (Pittenger et al., 1999; Liu et al., 2019; Shi et al., 2022). However, the therapeutic efficacy of BMSC-based approaches is still limited by the poor survival of transplanted cells in ischemic or injured tissues, where oxygen availability is often severely reduced. Enhancing BMSC tolerance to hypoxic stress may therefore be essential for improving the outcomes of stem cell-based tissue repair (Hu et al., 2008).

Hypoxia-induced apoptosis is a major contributor to the loss of transplanted BMSCs (Gabrielyan et al., 2020; Bornes et al., 2015). Sustained oxygen deprivation promotes reactive oxygen species (ROS) accumulation, mitochondrial dysfunction, and activation of pro-apoptotic pathways, ultimately reducing cell survival after transplantation (Wang et al., 2008; Ham and Raju, 2017). However, the signaling events that link hypoxic stress to apoptotic activation in BMSCs are not fully understood.

Hypoxia-inducible factor 1-alpha (HIF-1α) is a central mediator of cellular responses to oxygen deprivation, but its functional output is highly context-dependent (Schofield and Ratcliffe, 2004; Majmundar et al., 2010). Under mild-to-moderate physiological hypoxia (1%–7% O_2_), transient HIF-1α activation triggers adaptive responses that promote BMSCs survival, migration, and angiogenic capacity through mechanisms including the HIF-1α-GRP78-Akt axis and HIF-1α-dependent upregulation of C-X-C chemokine receptor type 4 (CXCR4) and Matrix metallopeptidase 9 (MMP9) (Wang et al., 2008; Zhao et al., 2007; Datta Chaudhuri et al., 2020; Najafi and Sharifi, 2013). Indeed, hypoxic preconditioning is widely used as a strategy to improve the post-transplantation survival and therapeutic efficacy of MSCs in ischemic tissues (Suzuki et al., 2001; Schmid et al., 2004; Zheng et al., 2019). However, under severe or sustained hypoxic stress—such as that encountered in pathological ischemia or modeled by high-dose CoCl_2_—excessive HIF-1α accumulation can overwhelm adaptive capacity and promote oxidative damage, mitochondrial dysfunction, and apoptosis (Wang et al., 2008; Ham and Raju, 2017). Yes-associated protein 1 (YAP), a major downstream effector of the Hippo pathway, also regulates stress-related cell fate decisions, including survival and apoptosis (Zhao et al., 2007; Ma et al., 2019; Sadri et al., 2024; Li et al., 2023; Ibar and Irvine, 2020). Recent studies suggest that HIF-1α and YAP may interact functionally under hypoxic conditions, though the direction of this interaction appears to depend on the cellular context and stress intensity (Chang et al., 2023; Wang et al., 2020; Tocci et al., 2023; Zhang et al., 2018). Notably, Wang et al. reported that HIF-1α/YAP activation protects rat BMSCs against compression-induced apoptosis in the degenerative disc microenvironment (Wang et al., 2020), suggesting a protective role for this axis under specific conditions. However, whether this pathway contributes to hypoxia-induced apoptosis under more severe hypoxic stress in BMSCs remains unclear.

Prodigiosin is a natural tripyrrole pigment produced by *Serratia marcescens* and related microorganisms, with reported antimicrobial, antitumor, antioxidant, and cytoprotective activities (Montaner and Pérez-Tomás, 2003; Araújo et al., 2022; Kim et al., 2023; Han et al., 2021; Zhao et al., 2022; Islan et al., 2022). Because ROS accumulation contributes substantially to hypoxia-induced apoptosis, Prodigiosin may help protect BMSCs from hypoxic injury (Chang et al., 2011). However, whether Prodigiosin can preserve BMSC survival under pathological hypoxia, and whether this effect involves HIF-1α/YAP-associated signaling, remains unclear.

In this study, we used a CoCl_2_-induced chemical hypoxia model (Yang et al., 2022) to test whether Prodigiosin protects BMSCs from hypoxia-induced apoptosis. We also examined whether HIF-1α/YAP-associated signaling is involved in this protective effect. Our results show that Prodigiosin reduces hypoxia-induced apoptosis in BMSCs, suggesting a potential approach to improve stem cell survival in hypoxic microenvironments.

## Materials and methods

### Cell isolation and culture

Mouse bone marrow-derived BMSCs were obtained from Cyagen Biosciences (Suzhou) Inc. (Cat. No. MUBMX-01001). The cells were cultured in DMEM (Gibco, United States) supplemented with 10% fetal bovine serum (FBS; Gibco, United States) and 1% penicillin/streptomycin (Gibco, United States) at 37 °C in a humidified incubator with 5% CO_2_. The culture medium was refreshed every 2–3 days. Upon reaching 80%–90% confluence, cells were passaged, and passage-2 cells were used for all subsequent experiments.

### Flow cytometric phenotyping of BMSCs

Passage-2 BMSCs were collected for phenotypic characterization. Cells were washed with PBS containing 1% bovine serum albumin and incubated with fluorophore-conjugated anti-CD90 (FITC) and anti-CD45 (PE) antibodies for 30 min at 4 °C in the dark. Unstained and isotype controls were used for gating. After incubation, the cells were washed and resuspended in PBS for flow cytometric analysis (BD Biosciences, San Jose, CA, United States). Data were analyzed using FlowJo v10.0 software (Tree Star Inc., Ashland, OR, United States).

### Induction of chemical hypoxia

Chemical hypoxia was induced using cobalt chloride (CoCl_2_; Sigma, United Kingdom). Before treatment, BMSCs were seeded and allowed to adhere for 24 h. The cells were then exposed to CoCl_2_ at final concentrations of 0, 1, 10, 50, 100, or 200 μM for 24 or 48 h. Cell viability and HIF-1α expression were subsequently assessed. Based on this screening, treatment with 100 μM CoCl_2_ for 24 h was selected as the standard chemical hypoxia condition for subsequent experiments.

### Drug treatment

Prodigiosin (MedChemExpress, HY-100711) was dissolved in DMSO to prepare stock solutions. BMSCs were treated with Prodigiosin at final concentrations of 10, 20, 40, 80, 100, or 200 nM in the presence of 100 μM CoCl_2_ for 24 or 48 h. Unless otherwise indicated, 20 nM Prodigiosin was used for subsequent experiments. BMSCs were co-treated with the YAP inhibitor Verteporfin (VP; MedChemExpress, HY-B0146) at 0.5 μM and CoCl_2_ under chemical hypoxia conditions.

### Cell viability assay

Cell viability was measured using a Cell Counting Kit-8 (CCK-8; Dojindo, Japan) according to the manufacturer’s instructions. BMSCs were seeded into 96-well plates at a density of 5 × 10^3^ cells/well and cultured for 24 h before treatment. For CoCl_2_ treatment, cells were exposed to 0, 1, 10, 50, 100, or 200 μM CoCl_2_ for 24 or 48 h. For Prodigiosin treatment, cells were incubated with 100 μM CoCl_2_ in the presence of 10, 20, 40, 80, 100, or 200 nM Prodigiosin for 24 or 48 h. After treatment, 10 μL CCK-8 reagent was added to each well containing 100 μL culture medium. Plates were incubated at 37 °C for 2 h in the dark. Absorbance at 450 nm was measured using a microplate reader (BioTek, United States). Cell viability was normalized to the untreated control group. All experiments were performed in triplicate.

### Annexin V/PI apoptosis assay

Cell apoptosis was assessed using an Annexin V–FITC/propidium iodide (PI) apoptosis detection kit (Beyotime Biotechnology, China, C1062 M) according to the manufacturer’s instructions. After the indicated treatments, both adherent and floating BMSCs were collected, washed twice with cold phosphate-buffered saline, and resuspended in 1× binding buffer at approximately cells/mL. A 100-μL cell suspension was incubated with 5 μL Annexin V–FITC and 5 μL PI for 15 min at room temperature in the dark. Subsequently, 400 μL binding buffer was added, and the samples were analyzed within 1 h using a flow cytometer (Beckman Coulter, United States). For flow cytometric analysis, cells were first gated on a FSC-A vs. SSC-A dot plot to exclude debris and cell aggregates. Doublet discrimination was then performed using FSC-A vs. FSC-H gating, where single cells were selected based on the FSC-A/FSC-H ratio. Apoptosis was analyzed on the singlet-gated population using Annexin V-FITC (FL1 channel) and PI (FL3 channel) fluorescence. Quadrant gates were set based on unstained and single-stained controls: viable cells (Annexin V^−^/PI^−^, lower left), early apoptotic cells (Annexin V^+^/PI^−^, lower right), late apoptotic/necrotic cells (Annexin V^+^/PI^+^, upper right), and necrotic cells (Annexin V^−^/PI^+^, upper left). A minimum of 10,000 events were acquired for each sample.

### Nuclei staining

BMSCs were seeded on glass coverslips placed in 24-well plates and treated as indicated. The cells were fixed with 4% paraformaldehyde and permeabilized with 0.1% Triton X-100. Nuclei were stained with DAPI (Beyotime Biotechnology, Cat# C1002) according to the manufacturer’s instructions. Fluorescence images were acquired using a fluorescence microscope (Olympus, Tokyo, Japan), and nuclear morphology was examined.

### ROS detection

Intracellular ROS levels were measured using a Tissue ROS Test Kit (Beijing Baiao Laibo, HR8821, China). After the indicated treatments, BMSCs cultured in 96-well plates were incubated with 200 μL of diluted dihydroethidium (DHE) probe (1:500) at 37 °C for 30 min in the dark. After incubation, fluorescence intensity was measured using a fluorescence microplate reader with excitation at 488–535 nm and emission at 610 nm.

### siRNA transfection

BMSCs at 50%–60% confluence were transfected with HIF-1α-targeting siRNA (Transgene, China) using a transfection reagent (Biodai, 11057, China) according to the manufacturer’s instructions. After 6 h, the medium was replaced with fresh complete medium. Twenty-four hours after transfection, the cells were treated with CoCl_2_ and/or Prodigiosin as indicated.

### Western blot analysis

After treatment, BMSCs were lysed using RIPA lysis buffer (Beyotime Biotechnology, Cat# P0013B) supplemented with protease and phosphatase inhibitor cocktail (Beyotime Biotechnology, Cat# P1046). Cell lysates were centrifuged at 13,000 rpm for 15 min at 4 °C, and the supernatants were collected for protein quantification using a BCA Protein Assay Kit (Beyotime Biotechnology, Cat# P0010S). Equal amounts of protein were separated by SDS-PAGE and transferred onto PVDF membranes (Millipore, United States). The membranes were blocked with 5% non-fat milk in TBST for 1 h at room temperature and incubated overnight at 4 °C with primary antibodies against HIF-1α (Abclonal Technology, A11945SP), GLUT1 (Abclonal Technology, A6982), active caspase-3 (Abclonal Technology, A11021), Bax (Abclonal Technology, A20227), YAP (Abclonal Technology, A19134), p-YAP Ser127 (Cell Signaling Technology, #13008), and GAPDH (Abclonal Technology, A19056). After incubation with HRP-conjugated secondary antibodies for 1 h at room temperature, protein bands were visualized using a BeyoECL Plus chemiluminescence kit (Beyotime Biotechnology, Cat# P0018S). Images were captured using a chemiluminescence imaging system (Tanon, China).

### Statistical analysis

Statistical analyses were performed using GraphPad Prism 7 software (GraphPad Software, San Diego, CA, United States). Data are presented as the mean ± standard error of the mean (SEM) from at least three independent experiments. Comparisons among multiple groups were analyzed using one-way or two-way analysis of variance (ANOVA) followed by Tukey’s *post hoc* test. Comparisons between two groups were performed using an unpaired two-tailed Student’s t-test. A value of P < 0.05 was considered statistically significant. Absence of statistical significance does not imply equivalence.

## Results

### CoCl_2_ establishes a hypoxia-like stress model and activates HIF-1α signaling in BMSCs

Before hypoxia induction, BMSCs were morphologically and phenotypically characterized. The cells exhibited a typical spindle-shaped morphology (Supplementary Figure S1A) and dual-staining flow cytometry showed that 95.53% of the cells were CD90^+^/CD45^-^ (with CD45^+^ cells representing only ∼1.1%), confirming the characteristic BMSC phenotype (Supplementary Figure S1B). The effects of CoCl_2_ concentration and exposure duration on cell viability were subsequently evaluated using the CCK-8 assay. After 24 h of treatment, cell viability remained largely unchanged at concentrations of 1–100 μM, whereas 200 μM CoCl_2_ significantly reduced cell viability (Figure 1A). To further characterize the dose-response relationship at intermediate concentrations, we compared cell viability between 50 μM and 100 μM CoCl_2_. At 24 h, 100 μM (96.5% ± 4.2%) versus 50 μM (98.1% ± 3.7%) showed no statistically significant difference (P = 0.645, unpaired two-tailed Student’s t-test). At 48 h, 100 μM (81.3% ± 5.1%) versus 50 μM (89.7% ± 4.6%) showed a trend toward reduction that did not reach statistical significance (P = 0.102). After 48 h, cell viability decreased at higher CoCl_2_ concentrations, with the most pronounced reduction observed at 200 μM (Figure 1B). HIF-1α protein expression increased following CoCl_2_ treatment, with significant increases observed at 100 and 200 μM compared with the untreated control (Figures 1C,D). Although 200 μM CoCl_2_ produced the highest HIF-1α expression, it was also associated with substantial cytotoxicity. In contrast, 100 μM CoCl_2_ effectively induced HIF-1α expression while maintaining relatively high cell viability at 24 h. Therefore, treatment with 100 μM CoCl_2_ for 24 h was selected for subsequent experiments. To further confirm that the stabilized HIF-1α was transcriptionally active, we examined the protein expression of GLUT1, a canonical HIF-1α target gene. Western blot analysis showed that GLUT1 protein levels also increased with increasing CoCl_2_ concentrations and were positively correlated with HIF-1α protein levels under the same conditions (Figure 1E). These results indicate that CoCl_2_ treatment activates a hypoxia-like transcriptional program rather than merely stabilizing HIF-1α protein. Together, these results indicate that 100 μM CoCl_2_ treatment for 24 h provides an appropriate condition for establishing a hypoxia-like stress model in BMSCs, characterized by HIF-1α induction without marked loss of cell viability.

**FIGURE 1:**
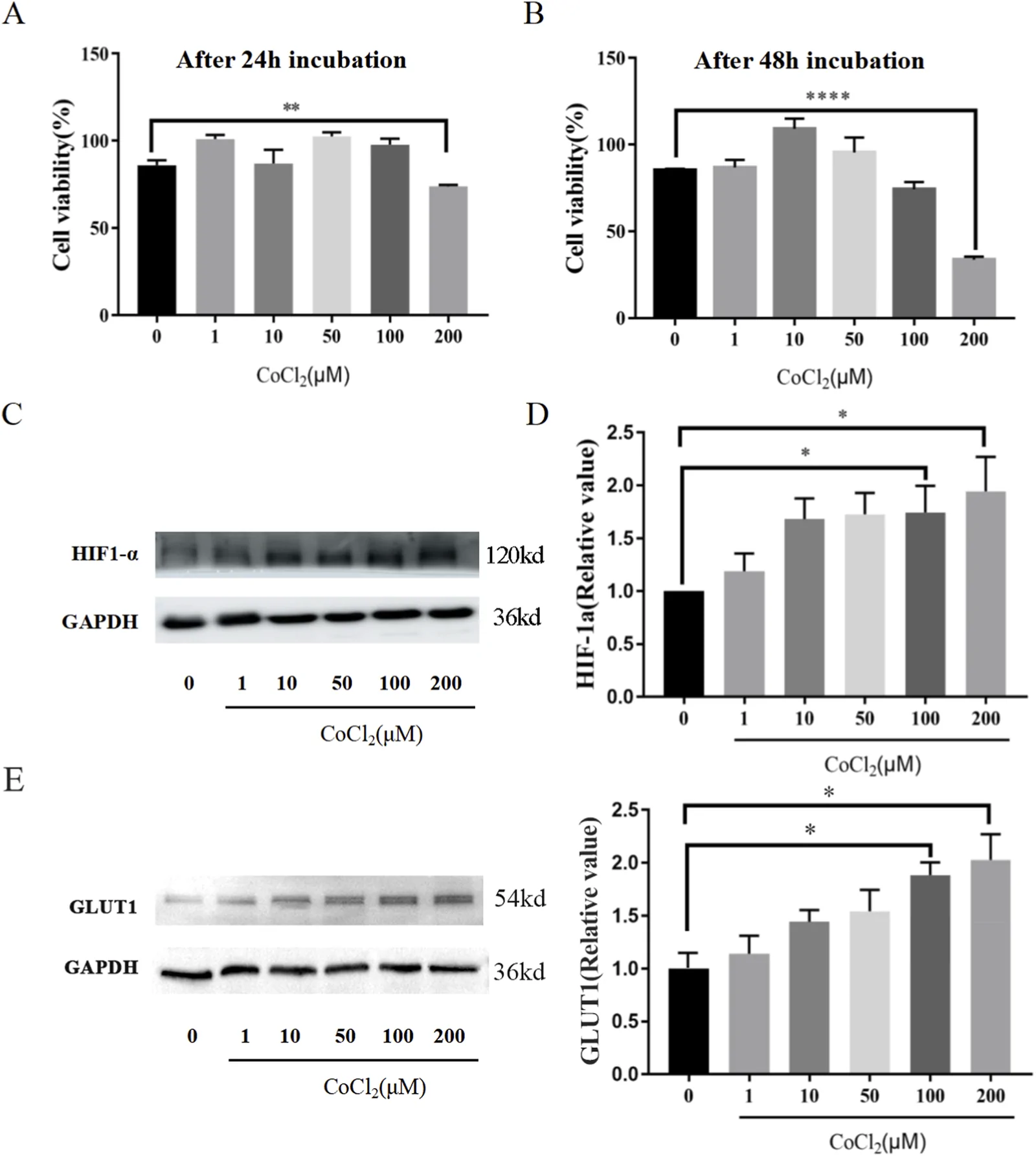
Establishment of a CoCl_2_-induced hypoxia-like stress model in BMSCs. **(A,B)** Cell viability of BMSCs treated with CoCl_2_ at increasing concentrations (0, 1, 10, 50, 100, and 200 μM) for 24 h or 48 h, as determined by CCK-8 assay. **(C,D)** Representative Western blot images and quantitative analysis of HIF-1α protein levels in BMSCs exposed to the indicated concentrations of CoCl_2_. **(E)** Western blot analysis and quantification of GLUT1 protein levels in BMSCs treated with increasing concentrations of CoCl_2_. Data are presented as mean ± SEM from three independent experiments. *P < 0.05, **P < 0.01, ****P < 0.0001 vs. control group. For intermediate concentration comparisons: 100 μM vs. 50 μM at 24 h, P = 0.645; at 48 h, P = 0.102 (unpaired two-tailed Student’s t-test).

### Prodigiosin attenuates hypoxia-induced cytotoxicity in BMSCs

To evaluate the effect of Prodigiosin on hypoxia-induced injury, BMSCs were treated with CoCl_2_ (100 μM) in the presence of increasing concentrations of Prodigiosin (10–200 nM), and cell viability was assessed using a CCK-8 assay (Figures 2A,B). CoCl_2_ treatment markedly reduced BMSC viability at both 24 h and 48 h, confirming the establishment of hypoxia-induced cytotoxicity. At 24 h, Prodigiosin at 20 nM significantly restored cell viability, whereas 10 nM showed only a modest effect and higher concentrations did not further enhance viability. At 48 h, 10 nM Prodigiosin significantly improved cell viability, while concentrations of 20 nM and above failed to provide protection and were associated with reduced viability. These findings indicate that Prodigiosin protects BMSCs against CoCl_2_-induced injury within a narrow concentration window, with the optimal protective concentration depending on exposure duration.

**FIGURE 2:**
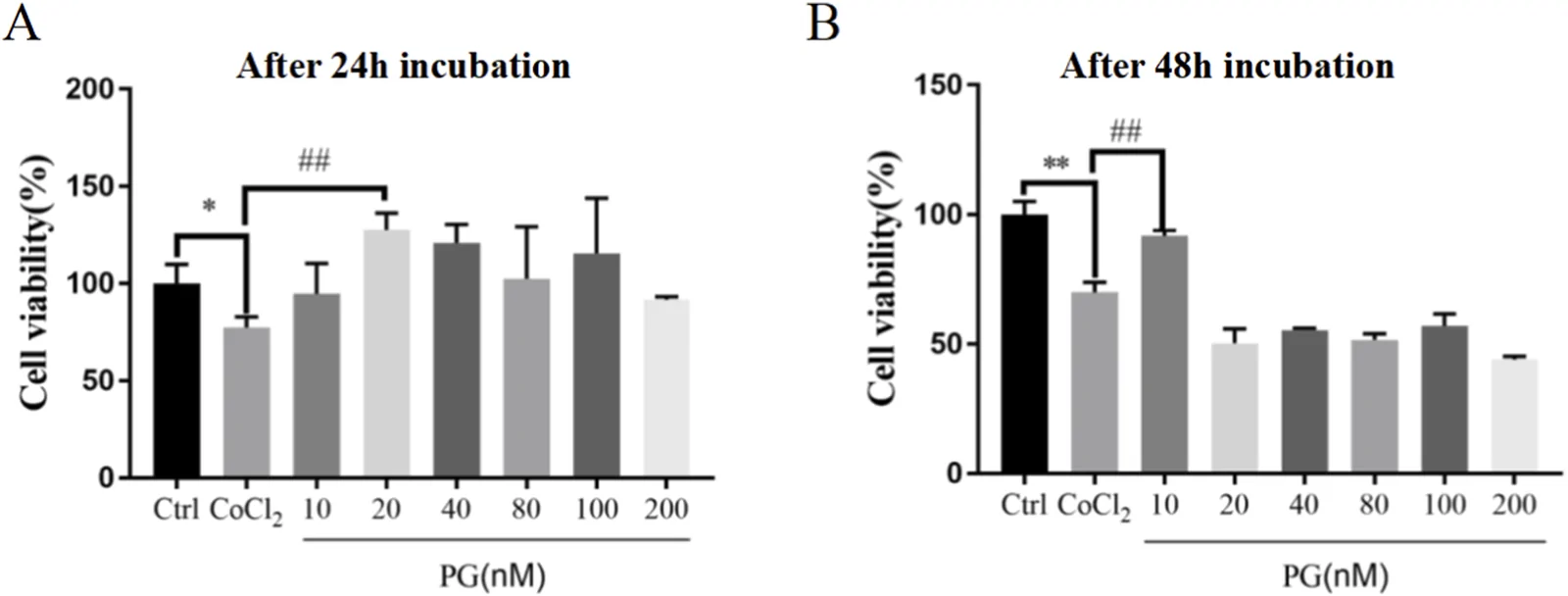
Prodigiosin alleviates CoCl_2_-induced cytotoxicity in BMSCs. BMSCs were treated with 100 μM CoCl_2_ in the presence of increasing concentrations of Prodigiosin (10–200 nM) for 24 h **(A)** and 48 h **(B)**. Cell viability was assessed using a CCK-8 assay. CoCl_2_ treatment significantly reduced BMSC viability. Data are presented as the mean ± SEM (n = 3). *P < 0.05 and **P < 0.01 vs. control group; ##P < 0.01 vs. CoCl_2_ group.

### Prodigiosin attenuates hypoxia-induced apoptotic injury in BMSCs

To further evaluate the protective effect of Prodigiosin under hypoxic stress, apoptosis was assessed by Annexin V/PI flow cytometry, together with DAPI staining and intracellular ROS measurement. While the CCK-8 assay was used as an initial screening tool for cell viability, Annexin V/PI flow cytometry served as the gold-standard orthogonal assay for apoptosis-specific detection. This distinction is important because CCK-8 measures mitochondrial dehydrogenase activity and reflects overall metabolic activity, whereas Annexin V/PI specifically detects apoptosis. CoCl_2_ treatment significantly increased the total apoptosis rate in BMSCs, which was markedly reduced by 20 nM Prodigiosin (Figures 3A,B). Consistently, DAPI staining revealed prominent nuclear condensation and fragmentation following CoCl_2_ exposure, whereas these apoptotic morphological changes were clearly alleviated by Prodigiosin (Figures 3C,D). To determine whether CoCl_2_-induced apoptosis was associated with oxidative stress, intracellular ROS levels were measured using the DHE fluorescent probe. As shown in Figure 3E, CoCl_2_ treatment significantly increased ROS production in BMSCs compared to the untreated control group. However, co-treatment with Prodigiosin (20 nM) markedly attenuated CoCl_2_-induced ROS generation, reducing the fluorescence intensity to near-control levels. Together, these results demonstrate that Prodigiosin attenuates hypoxia-induced oxidative stress and apoptosis in BMSCs, supporting its protective effect under hypoxic conditions.

**FIGURE 3:**
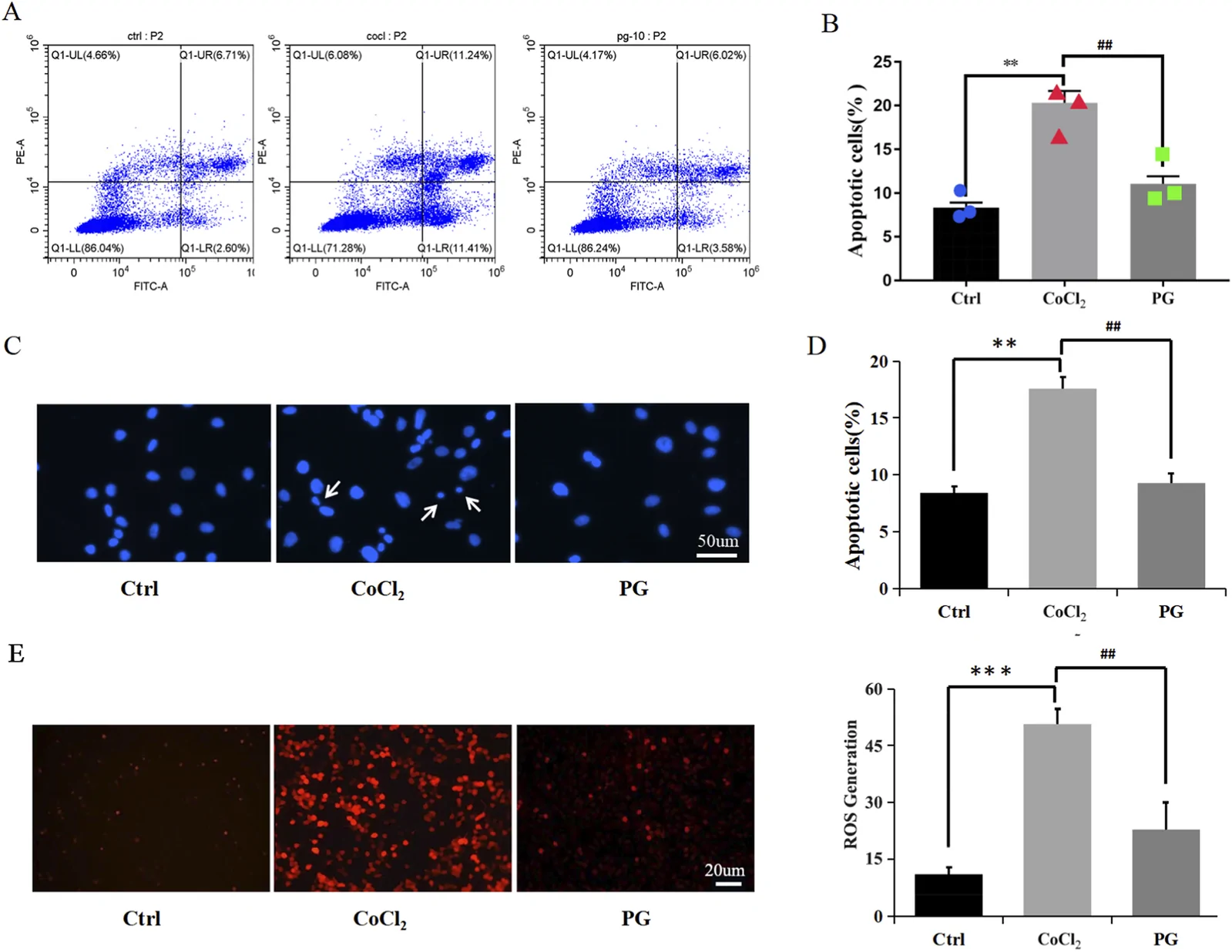
Prodigiosin suppresses CoCl_2_-induced apoptosis and oxidative stress in BMSCs. BMSCs were treated with 100 μM CoCl_2_ and/or 20 nM Prodigiosin (PG) for 24 h. **(A)** Representative Annexin V-FITC/PI flow cytometry plots of BMSCs in the Ctrl, CoCl_2_, and CoCl_2_+PG groups. The four quadrants represent viable cells (LL), early apoptotic cells (LR), late apoptotic/necrotic cells (UR), and necrotic cells (UL). **(B)** Quantitative analysis of apoptotic cells determined by flow cytometry. **(C)** Representative fluorescence microscopy images of DAPI-stained nuclei. Arrows indicate cells with condensed or fragmented apoptotic nuclei. Scale bar = 50 μm. **(D)** Quantitative analysis of apoptotic cells based on DAPI nuclear morphology. **(E)** Intracellular ROS levels measured by DHE fluorescent probe. Representative fluorescence intensity (left) and quantitative analysis of relative fluorescence intensity (right). Data are presented as the mean ± SEM from three independent experiments (n = 3). **P < 0.01 and ***P < 0.001vs. control group; ##P < 0.01 vs. CoCl_2_ group.

### Prodigiosin suppresses HIF-1α signaling and apoptosis-related protein expression in hypoxic BMSCs

To examine the molecular changes associated with the anti-apoptotic effect of Prodigiosin, the expression of hypoxia- and apoptosis-related factors was evaluated at the mRNA and protein levels. RT-qPCR analysis showed that CoCl_2_ treatment significantly increased the mRNA expression of HIF-1α and active caspase-3, whereas co-treatment with Prodigiosin significantly reduced the expression of both genes compared with CoCl_2_ treatment alone (Figures 4A,B). Western blot analysis further showed that CoCl_2_ treatment increased the protein levels of HIF-1α, active caspase-3, and Bax. These increases were significantly attenuated by Prodigiosin co-treatment (Figures 4C,D).

**FIGURE 4:**
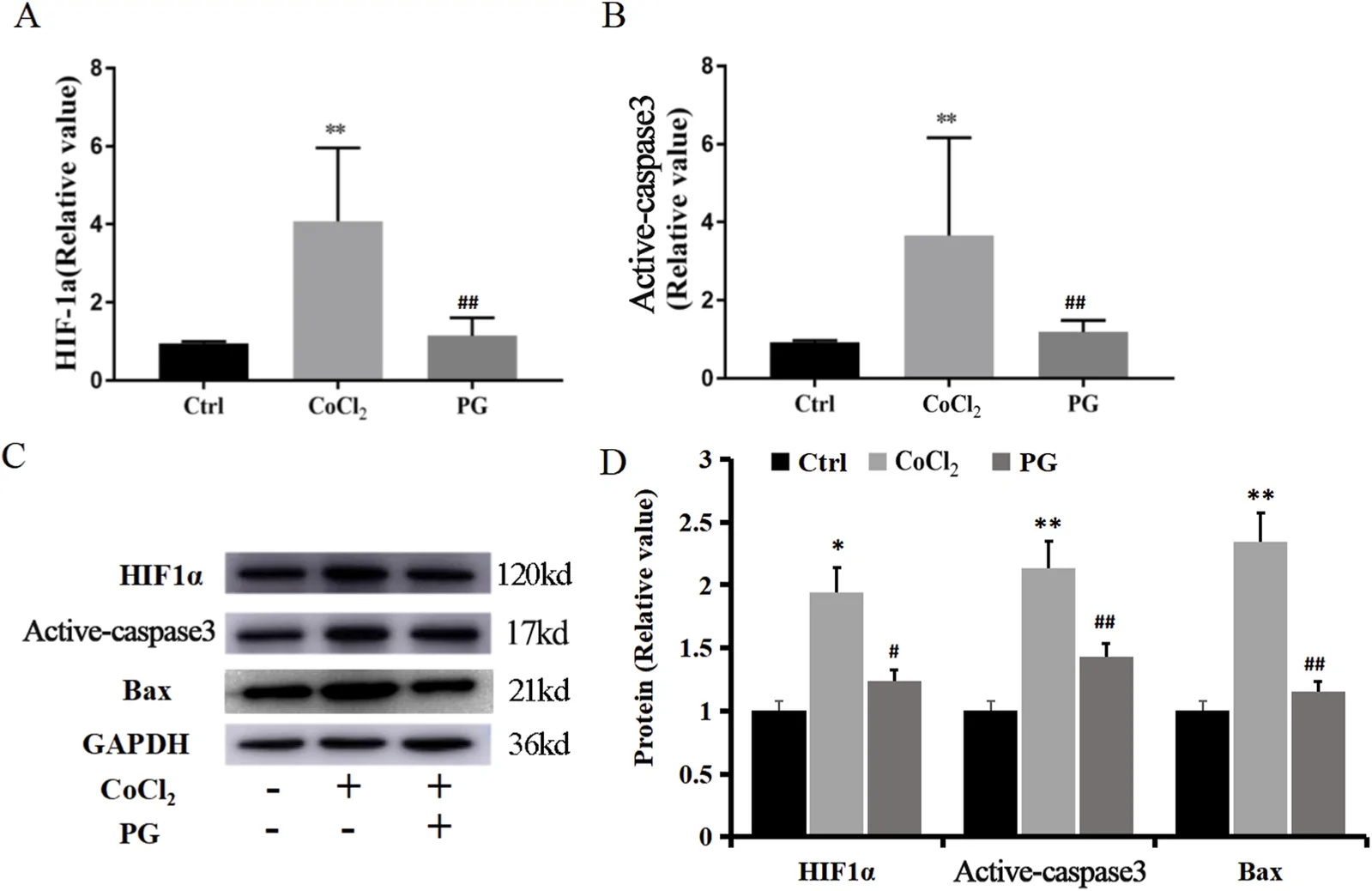
Prodigiosin suppresses HIF-1α signaling and apoptosis-related protein expression in hypoxic BMSCs. BMSCs were treated with 100 μM CoCl_2_ and/or 20 nM Prodigiosin for 24 h. **(A,B)** RT-qPCR analysis of HIF-1α **(A)** and active caspase3 **(B)** mRNA expression in BMSCs treated with CoCl_2_ alone or in combination with Prodigiosin. Data are presented as relative values normalized to the control group (Ctrl). **(C,D)** Representative Western blot images showing the protein levels of HIF-1α (∼120 kDa), active caspase3 (∼17 kDa), and Bax (∼21 kDa), GAPDH (∼36 kDa) was used as the loading control. (*P < 0.05 and **P < 0.01 vs. control group, #p < 0.05 and ##p < 0.01 vs. CoCl_2_ group). Data are expressed as mean ± SEM.

Together, these results demonstrate that Prodigiosin attenuates the CoCl_2_-induced upregulation of hypoxia- and apoptosis-related molecular markers, consistent with its anti-apoptotic effects observed at the cellular level.

### Prodigiosin attenuates hypoxia-induced apoptosis in BMSCs via midulation of HIF-1α/YAP-associated signaling

To examine the molecular changes associated with Prodigiosin treatment and YAP inhibition, the protein levels of HIF-1α, YAP1, p-YAP1, active caspase-3, and Bax were assessed by Western blotting. CoCl_2_ treatment significantly increased the expression of HIF-1α, YAP1, p-YAP1, active caspase-3, and Bax compared with the control group. Co-treatment with Prodigiosin significantly reduced YAP1, p-YAP1, active caspase-3, and Bax levels, whereas no significant reduction in HIF-1α expression was observed (Figures 5A,B). Treatment with the YAP inhibitor Verteporfin similarly reduced YAP1, p-YAP1, active caspase-3, and Bax expression without significantly decreasing HIF-1α levels. Flow-cytometric analysis further showed that CoCl_2_ treatment significantly increased the proportion of apoptotic cells. Prodigiosin markedly reduced CoCl_2_-induced apoptosis, while Verteporfin also partially attenuated the increase in apoptosis (Figures 5C,D). Together, these results indicate that both Prodigiosin and Verteporfin attenuate CoCl_2_-induced apoptotic responses and are associated with reduced YAP1 and p-YAP1 expression. However, these results alone do not establish a causal HIF-1α/YAP signaling axis.

**FIGURE 5:**
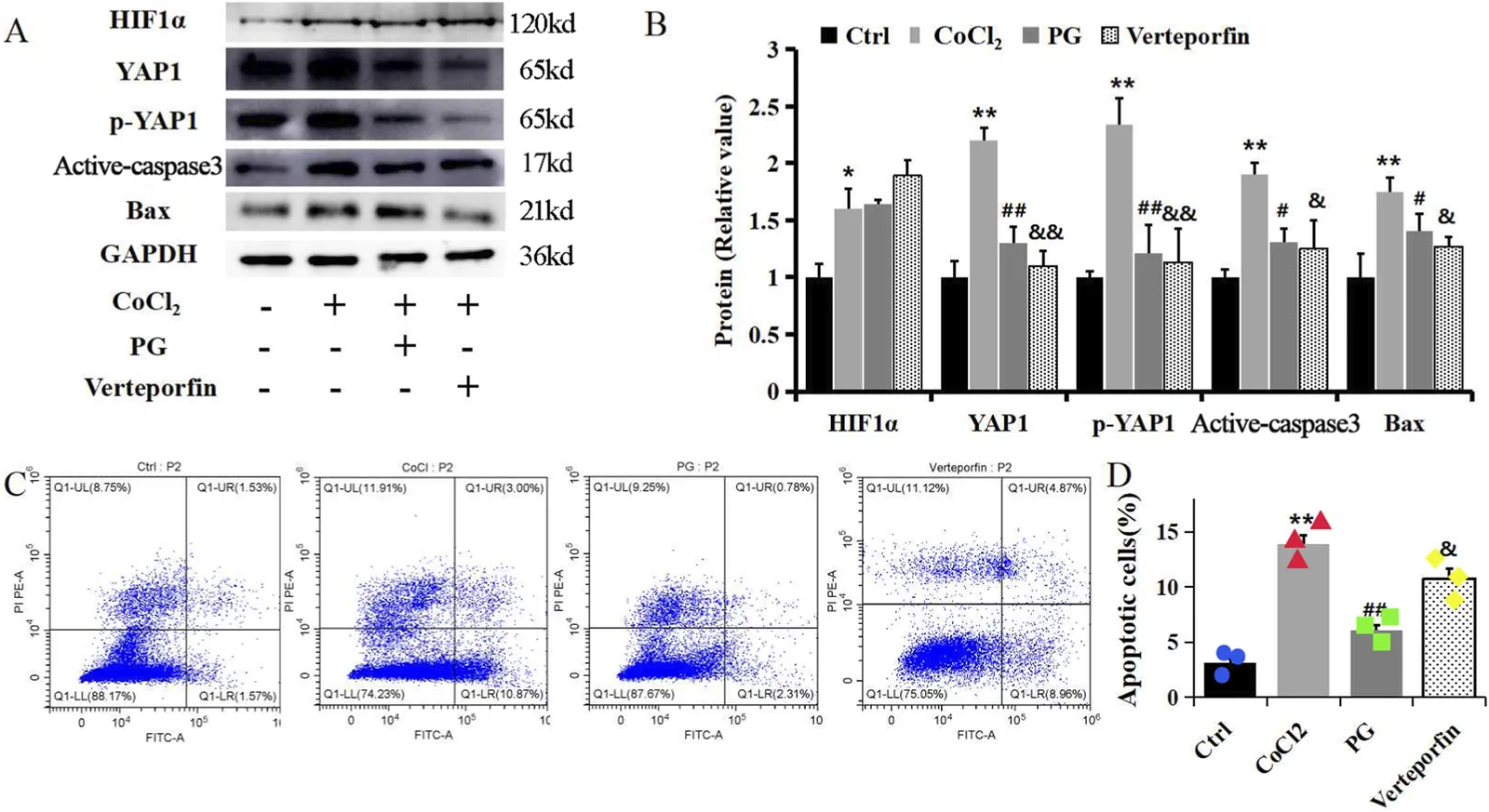
Prodigiosin attenuates hypoxia-induced apoptosis in BMSCs via modulation of the HIF-1α/YAP-associated signaling. BMSCs were treated with 100 μM CoCl_2_, 20 nM Prodigiosin and/or 0.5 μM Verteporfin for 24 h. **(A,B)** Representative Western blot images and quantitative analysis of HIF-1α, YAP1, p-YAP1, active caspase-3, and Bax protein levels in BMSCs. GAPDH served as the loading control. **(C,D)** Representative flow cytometry dot plots (Annexin V-FITC/PI staining) and quantification of total apoptotic cell percentages in BMSCs across treatment groups. All data are presented as the mean ± SEM from three independent experiments (n = 3). *P < 0.05 and **P < 0.01 vs. control group; ^#^P < 0.05 and ^##^P < 0.01 vs. CoCl_2_ group.

### HIF-1α mediates the anti-apoptotic effect of prodigiosin under hypoxic conditions

To directly test whether the cytoprotective effect of Prodigiosin is HIF-1α-dependent, we reasoned that genetic silencing of HIF-1α should phenocopy Prodigiosin treatment if the two share a common mechanistic target. siRNA-mediated knockdown of HIF-1α was therefore performed in parallel with Prodigiosin treatment in CoCl_2_-challenged BMSCs. At the molecular level, both interventions comparably suppressed HIF-1α protein expression and accompanied by changes in YAP phosphorylation, as reflected by reduced total YAP and restored p-YAP (Ser127) levels; concurrent reductions in active caspase-3 and Bax further indicated attenuation of apoptotic signaling (Figures 6A,B). At the phenotypic level, Annexin V/PI flow cytometry confirmed that both Prodigiosin and siHIF-1α significantly reduced CoCl_2_-induced apoptosis to a comparable extent (Figures 6C,D). The convergence of molecular and phenotypic outcomes across two independent modes of HIF-1α inhibition—pharmacological and genetic—supports the conclusion that Prodigiosin exerts its anti-apoptotic effect at least in part through modulation of the HIF-1α/YAP-assoctated signaling.

**FIGURE 6:**
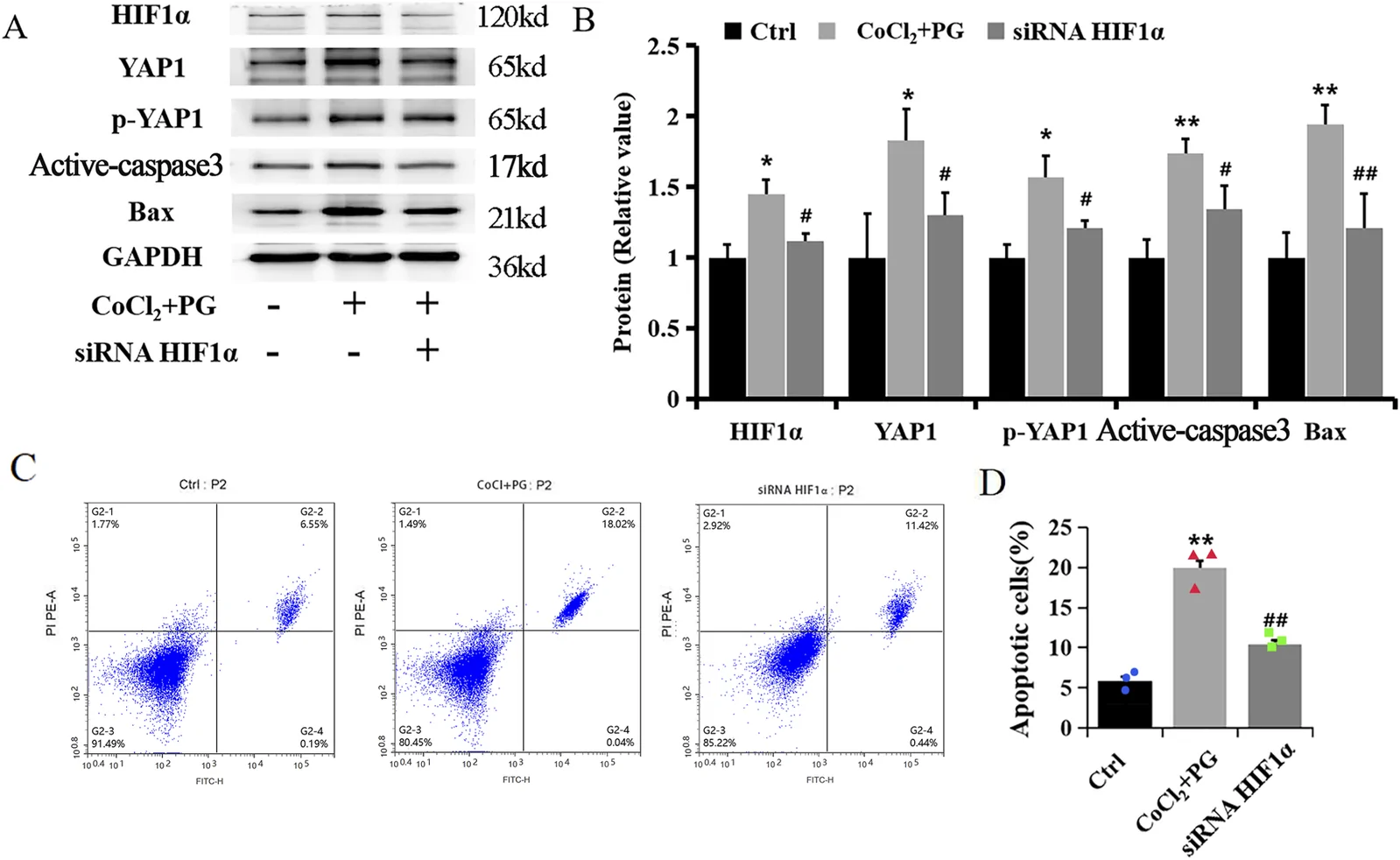
The anti-apoptotic effect of Prodigiosin is phenocopied by HIF-1α silencing in hypoxic BMSCs. BMSCs were treated with 100 μM CoCl_2_ and/or 20 nM Prodigiosin for 24 h. **(A)** Representative Western blot images of YAP, p-YAP HIF-1a, Caspase3 and Bax with treatment of Prodigiosin and si-HIF-1α under hypoxia condition. **(B)** Densitometric analysis of relative protein level (normalized to GAPDH). **(C,D)** Apoptosis was assessed by Annexin V/PI staining and flow cytometry. Representative plots **(C)** and quantitative analysis of the total apoptotic rate. All data are presented as the mean ± SEM from three independent experiments (n = 3). *P < 0.05 and **P < 0.01 vs. control group; ^#^P < 0.05 and ^##^P < 0.01 vs. CoCl_2_ group.

In summary, this study identifies HIF-1α as a key mediator of hypoxia-induced apoptosis in BMSCs and demonstrates that Prodigiosin confers cytoprotection by targeting this pathway.

Under hypoxic conditions modeled by CoCl_2_, HIF-1α accumulates and is accompanied by dysregulation of YAP-associated signaling and enhanced downstream transcriptional activity. These molecular alterations are associated with upregulation of Bax and active caspase-3, ultimately promoting BMSC apoptosis. Prodigiosin effectively attenuates this process by suppressing HIF-1α accumulation, which is accompanied by normalization of YAP-associated signaling and reduced apoptotic marker expression, thereby improving BMSC survival under hypoxic conditions. The dependence of this protective effect on HIF-1α is further supported by the finding that siRNA-mediated HIF-1α silencing phenocopies the anti-apoptotic actions of Prodigiosin.

Taken together, these findings highlight the HIF-1α/YAP axis as a potential therapeutic target in hypoxia-injured BMSCs and suggest that Prodigiosin warrants further investigation as a cytoprotective strategy in ischemia-related stem cell therapies.

## Discussion

In this study, we showed that Prodigiosin protects BMSCs against hypoxia-induced apoptosis and that this effect is associated with inhibition of HIF-1α/YAP-related signaling. In a CoCl_2_-induced chemical hypoxia model, hypoxic stress reduced BMSC viability, increased intracellular ROS accumulation, and activated apoptotic signaling, as indicated by increased Bax and active caspase-3 expression. Prodigiosin markedly alleviated these cellular and molecular changes. At the molecular level, Prodigiosin reduced HIF-1α accumulation, partially restored the p-YAP/YAP ratio, and decreased the expression of YAP target genes. In addition, both pharmacological inhibition of YAP and knockdown of HIF-1α attenuated CoCl_2_-induced apoptotic responses. These results suggest that HIF-1α accumulation and YAP activation contribute to hypoxia-induced BMSC injury and that Prodigiosin counteracts this process, at least in part, by modulating HIF-1α/YAP-associated signaling.

In the present study, HIF-1α accumulation under severe CoCl_2_-induced hypoxia (100 μM, 24 h) was accompanied by elevated ROS and apoptosis, contrasting with its protective role under mild hypoxia. This context-dependent switch is consistent with previous reports that severe hypoxia induces apoptosis, whereas moderate hypoxia can be protective (Pittenger et al., 1999; Liu et al., 2019; Hu et al., 2008). This is consistent with the finding that the duration and severity of hypoxia determine whether p53-mediated outcomes favor survival or apoptosis (Humpton and Vousden, 2016). Although CoCl_2_ may exert cobalt-specific effects, several observations support a HIF-1α-mediated component: HIF-1α induction preceded apoptosis (Figures 1C,D vs; Figures 3A,B); HIF-1α knockdown phenocopied the anti-apoptotic effect of Prodigiosin (Figure 6); and the YAP inhibitor Verteporfin attenuated apoptosis independent of CoCl_2_ concentration. These findings suggest that under the severe hypoxic conditions used here, HIF-1α contributes to BMSCs injury rather than protection.

A key finding of this study is that YAP contributes to the apoptotic response of BMSCs under hypoxic stress and may act in functional association with HIF-1α signaling. Although YAP is commonly linked to proliferation, mechanotransduction, and stem cell fate regulation, its output is highly context-dependent (Zhao et al., 2007; Ma et al., 2019; Sadri et al., 2024; Li et al., 2023; Ibar and Irvine, 2020). In our CoCl_2_-induced model, reduced p-YAP/YAP ratio and increased YAP target gene expression indicated enhanced YAP activity, which coincided with elevated Bax and active caspase-3 expression. Verteporfin-mediated YAP inhibition (Liu-Chittend et al., 2012) reduced apoptosis-related markers and attenuated cell death, supporting a pro-apoptotic role for YAP in this setting. Thus, in hypoxia-injured BMSCs, YAP activation may amplify apoptotic signaling rather than simply promoting survival.

Reconciling our findings with the protective role of HIF-1α/YAP reported by Wang et al. (2020). Although our finding that HIF-1α promotes apoptosis in CoCl_2_-treated BMSCs appears to contrast with the report by Wang et al. (2020), who showed that HIF-1α/YAP activation protects BMSCs against compression-induced apoptosis, this discrepancy may be explained by contextual differences. Wang et al. used mechanical compression combined with moderate hypoxia, a milder stress that likely engages adaptive HIF-1α signaling, whereas our 100 μM CoCl_2_ model produces severe chemical hypoxia with direct oxidative damage that may exceed the capacity of HIF-1α-mediated adaptation; this is consistent wiht the view that HIF-1α shifts from protective to injurious as stress intensity increases (Shi et al., 2022; Hu et al., 2008; Bornes et al., 2015). In addition, hypoxic preconditioning studies typically use brief, intermittent hypoxia (1–4 h), whereas our 24-h continuous exposure represents sustained stress that may exhaust protective mechanisms. Prolonged hypoxia has been shown to trigger mitochondrial dysfunction and active caspase-3 activation, tipping the balance toward apoptosis (Ham and Raju, 2017; Datta Chaudhuri et al., 2020; Schmid et al., 2004; Zheng et al., 2019). This is supported by evidence that prolonged exposure to hypoxia leads to mitochondrial malfunction and caspase-3 activation, a crucial factor in apoptosis. Furthermore, Wang et al.'s compression model and our CoCl_2_ model invove distinct concurrent stressors (mechanical vs. oxidative), which may recruit different downstream effectors and co-factors and lead to opposing outcomes (Pittenger et al., 1999; Hu et al., 2008). Thus, HIF-1α may function as a ‘stress rheostat’ whose output depends on the nature, intensity, and duration of the stress stimulus (Gabrielyan et al., 2020; Bornes et al., 2015). Our results therefore complement, rather than contradict, the findings of Wang et al. by demonstrating that under severe, purely hypoxic stress (without mechanical compression), the same HIF-1α/YAP-associated signaling can promote apoptosis rather than survival.

The HIF-1α knockdown experiments further support a functional association between HIF-1α and YAP in this severe hypoxia model. Silencing HIF-1α not only reduced apoptotic injury but also attenuated YAP-associated signaling changes, suggesting that HIF-1α and YAP are functionally associated, with HIF-1α potentially acting upstream of YAP; however, direct regulation requires further investigation under the severe CoCl_2_-induced hypoxic stress conditions used in this study. The similarity between the effects of HIF-1α knockdown and Prodigiosin treatment suggests that Prodigiosin protects BMSCs, at least in part, by suppressing HIF-1α accumulation and associated YAP activation. However, because rescue experiments using HIF-1α overexpression or constitutively active YAP were not performed, this pathway should be interpreted as a strongly supported mechanistic association rather than a fully established linear cascade.

Another important implication of this study is the context-dependent biological activity of Prodigiosin. Previous studies have mainly focused on its antimicrobial and antitumor properties (Montaner and Pérez-Tomás, 2003; Araújo et al., 2022; Kim et al., 2023; Han et al., 2021; Zhao et al., 2022; Islan et al., 2022), whereas our findings suggest that Prodigiosin can also exert cytoprotective effects in normal stem cells under hypoxia-like stress. This apparent dual activity may depend on cell type, stress intensity, and dosage (Chang et al., 2011). Notably, the protective effect of Prodigiosin was observed within a relatively narrow concentration range, and higher concentrations did not provide additional benefit. This dose-dependent profile indicates that careful optimization will be necessary before Prodigiosin can be considered as a preconditioning agent to improve BMSC survival in ischemic or poorly vascularized tissue environments.

Notably, the optimal protective concentration that the optimal protective concentration of Prodigiosin shifted from 20 nM at 24 h to 10 nM at 48 h, with higher concentrations showing reduced protection at the later time point. This biphasic response may reflect that this biphasic response reflects two competing activities operating on different time scales: at lower concentrations (10–20 nM), Prodigiosin acts as a rapid ROS scavenger that attenuates CoCl_2_-induced oxidative stress, whereas prolonged exposure and higher concentrations progressively engage its well-documented antiproliferative and pro-apoptotic activities (Montaner and Pérez-Tomás, 2003; Araújo et al., 2022; Kim et al., 2023; Han et al., 2021; Zhao et al., 2022; Islan et al., 2022), eventually overriding its protective effects and explaining why 20 nM Prodigiosin, protective at 24 h, became detrimental at 48 h. Notably, Prodigiosin exhibits significantly lower toxicity in normal cells than in cancerous counterparts; however, the 48 h reversal at 20 nM likely represents the threshold at which its intrinsic antiproliferative activity begins to manifest in BMSCs, defining a narrow therapeutic window (10–20 nM, ≤24 h) for ROS scavenging and apoptosis suppression, beyond which cytotoxicity dominates—consistent with the hormetic dose-responses observed for many cytoprotective agents. Furthermore, the cytoprotective effect observed here is mechanistically distinct from Prodigiosin’s anticancer activity (Montaner and Pérez-Tomás, 2003; Araújo et al., 2022; Kim et al., 2023; Han et al., 2021; Zhao et al., 2022; Islan et al., 2022): in cancer cells, it induces apoptosis via ROS generation and caspase activation, whereas in stressed BMSCs at low concentrations, it acts as an ROS scavenger that attenuates oxidative damage. This context-dependent duality reflects that Prodigiosin’s biological output depends on cellular context and exposure conditions, extending its known biology by demonstrating a protective role in normal stem cells under pathological stress.

Several limitations should be acknowledged. First, this study used CoCl_2_ to establish a chemical hypoxia model. Although CoCl_2_ effectively stabilizes HIF-1α and induces hypoxia-like signaling, it does not fully reproduce the complexity of physical low-oxygen conditions and may introduce cobalt-specific effects (Yang et al., 2022; Vengellur and Lapres, 2004; Barton et al., 2025). Importantly, our dose-dependent analysis (Figure 1) showedno statistically significant difference in viability between 100 μM compared to 50 μM at 24 h (96.5% ± 4.2% vs. 98.1% ± 3.7%, P = 0.645, unpaired two-tailed Student’s t-test). However, this non-significant result does not demonstrate equivalence, and the small sample size (n = 3) limits the power to detect small differences. The selection of 100 μM CoCl_2_ was based on its ability to induce HIF-1α without marked cytotoxicity at 24 h and on its use in previous studies, not on a formal equivalence test. Nevertheless, 100 μM CoCl_2_ for 24 h represents a severe chemical hypoxic insult that should be distinguished from the mild-to-moderate physiological hypoxia (1%–7% O_2_) used in preconditioning studies; thus, our findings should be interpreted within this specific pathological stress context. Future studies should validate these findings using controlled low-oxygen culture systems or alternative non-metal hypoxia mimetics. Second, although our data show that Prodigiosin suppresses HIF-1α/YAP-associated signaling, its direct molecular target remains unknown. Third, the downstream transcriptional mechanisms by which YAP contributes to Bax and active caspase-3 upregulation, as well as whether HIF-1α directly regulates YAP, require further clarification through YAP nuclear localization analysis, TEAD-dependent transcriptional assays, co-immunoprecipitation, or rescue experiments. Finally, all experiments were performed *in vitro*, and *in vivo* studies are needed to determine whether Prodigiosin improves the survival and therapeutic efficacy of transplanted BMSCs in ischemic tissues.

In conclusion, this study provides evidence that Prodigiosin protects BMSCs from hypoxia-induced apoptosis, at least in part by modulating HIF-1α/YAP-associated signaling (Figure 7). These findings suggest that Prodigiosin warrants further investigation as a potential cytoprotective agent, although *in vivo* studies are needed to determine its translational potential for improving stem cell survival in ischemic tissues.

**FIGURE 7:**
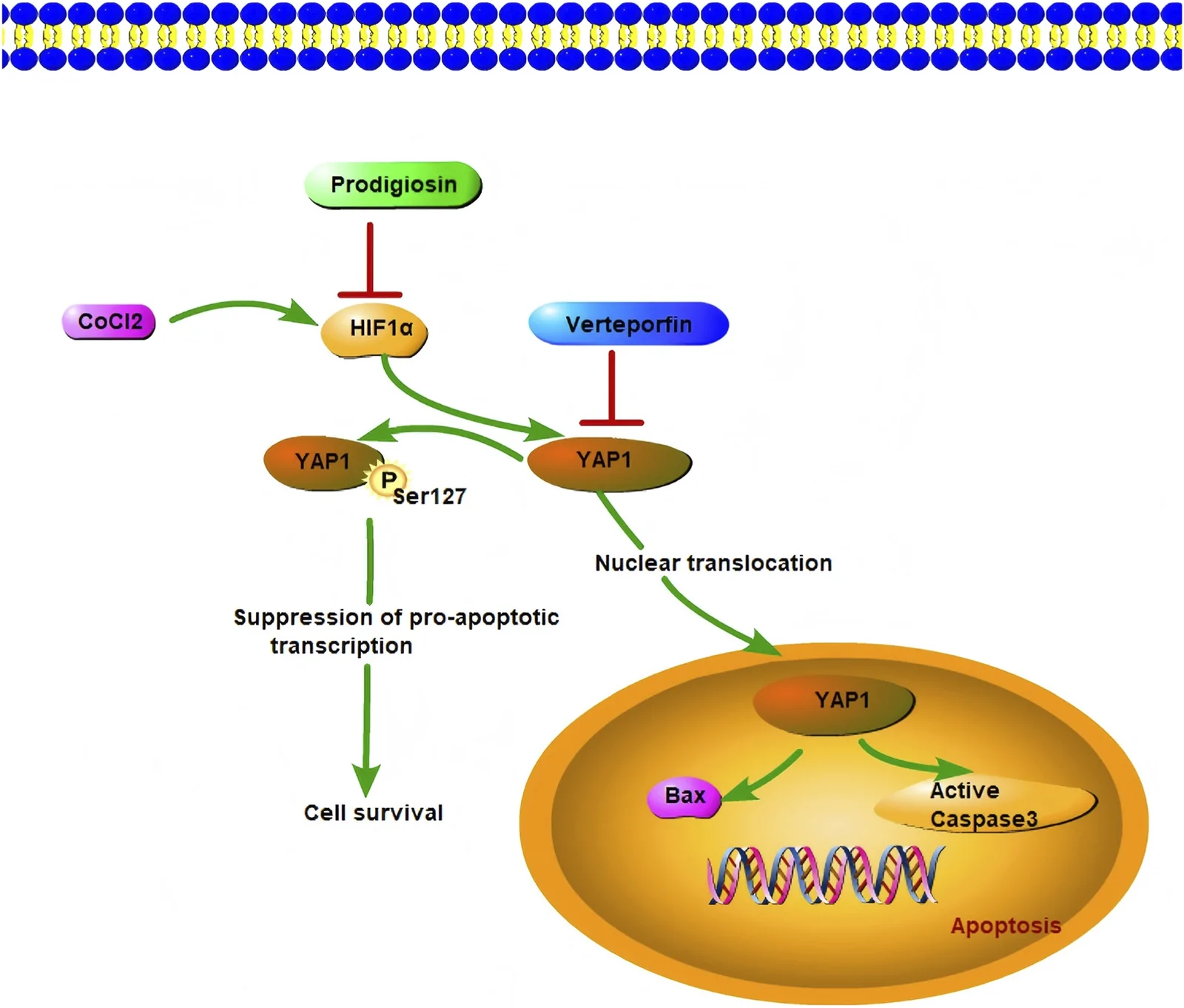
Proposed mechanism for the protective effect of Prodigiosin on BMSCs under hypoxic conditions. The schematic diagram illustrates the proposed signaling pathway. Under hypoxic conditions, HIF-1α protein is stabilized. This is accompanied by YAP dephosphorylation and nuclear translocation, and nuclear YAP upregulates pro-apoptotic effectors such as Bax and active caspase-3, ultimately resulting in BMSC apoptosis. Prodigiosin exerts its potent anti-apoptotic effect by inhibiting HIF-1α, thereby promoting YAP phosphorylation and cytoplasmic retention, blocking the downstream cell death signal and promoting cell survival. The direct regulatory relationship between HIF-1α and YAP remains to be established.

## Data Availability

The original contributions presented in the study are included in the article, further inquiries can be directed to the corresponding author.

## Supplementary material

SUPPLEMENTARY FIGURE S1 Morphological and phenotypic characterization of BMSCs. **(A)** Cells displayed a typical spindle-shaped morphology. **(B)** Flow cytometric analysis of BMSCs. Left: gating strategy (FSC-H vs SSC-H, G1 gate). Right: dual staining for CD90 (FITC) and CD45 (PE) within the G1 gate. The majority of cells (95.53%) were CD90+/CD45-, consistent with the characteristic BMSC phenotype. Unstained and isotype controls were used for gating.
